# Risk factor analysis for fast track protocol failure

**DOI:** 10.1186/1749-8090-8-47

**Published:** 2013-03-15

**Authors:** Arndt H Kiessling, Patrick Huneke, Christian Reyher, Tobias Bingold, Andreas Zierer, Anton Moritz

**Affiliations:** 1 Department of Thoracic and Cardiovascular Surgery, Johann Wolfgang Goethe University, Theodor Stern Kai 7, 60590, Frankfurt am Main, Germany; 2 Department of Anaesthesiology and Intensive Care, Johann Wolfgang Goethe University, Frankfurt am Main, Germany

**Keywords:** Fast-track, ICU, Cardiac surgery, Readmission, Outcome

## Abstract

**Background:**

The introduction of fast-track treatment procedures following cardiac surgery has significantly shortened hospitalisation times in intensive care units (ICU). Readmission to intensive care units is generally considered a negative quality criterion. The aim of this retrospective study is to statistically analyse risk factors and predictors for re-admission to the ICU after a fast-track patient management program.

**Methods:**

229 operated patients (67 ± 11 years, 75% male, BMI 27 ± 3, 6/2010-5/2011) with use of extracorporeal circulation (70 ± 31 min aortic crossclamping, CABG 62%) were selected for a preoperative fast-track procedure (transfer on the day of surgery to an intermediate care (IMC) unit, stable circulatory conditions, extubated). A uni- and multivariate analysis were performed to identify independent predictors for re-admission to the ICU.

**Results:**

Over the 11-month study period, 36% of all preoperatively declared fast-track patients could not be transferred to an IMC unit on the day of surgery (n = 77) or had to be readmitted to the ICU after the first postoperative day (n = 4). Readmission or ICU stay signifies a dramatic worsening of the patient outcome (mortality 0/10%, mean hospital stay 10.3 ± 2.5/16.5 ± 16.3, mean transfusion rate 1.4 ± 1,7/5.3 ± 9.1). Predicators for failure of the fast-track procedure are a preoperative ASA class > 3, NYHA class > III and an operation time >267 min ± 74. The significant risk factors for a major postoperative event (= low cardiac output and/or mortality and/or renal failure and/or re-thoracotomy and/or septic shock and/or wound healing disturbances and/or stroke) are a poor EF (OR 2.7 CI 95% 0.98-7.6) and the described ICU readmission (OR 0.14 CI95% 0.05-0.36).

**Conclusion:**

Re-admission to the ICU or failure to transfer patients to the IMC is associated with a high loss of patient outcome. The ASA > 3, NYHA class > 3 and operation time >267 minutes are independent predictors of fast track protocol failure.

## Background

Since open heart surgery became established in the 1950s, the sedation and prolonged ventilatory support of this patient population has been the undisputed standard practice. Prolonged ventilatory support was maintained at least until the morning of the first postoperative day until the hemodynamic, respiratory and coagulation physiological systems had stabilised completely. Particularly the first few hours after cardiac surgical interventions are regarded as a critical period for the occurrence of myocardial ischemia [1-4], which are frequently triggered by the hypothermic and hemodilution as a consequence of the extracorporeal circulation and the consecutive activation of the sympathetic nervous system [1,5]. Moreover, the extracorporeal circulation itself caused transient functional and metabolic damage to the myocardium, which consequently became even more susceptible to new onset ischemia [4,6]. An additional aspect of sedated patient oberservation was the adequate monitoring of potential complications such as uncontrollable hypertension, arrhythmias and the risk of postoperative hemorrhage [4]. In order to more effectively control these serious complications, the patients should be kept under sedation in stable, easily supervisable conditions until the occurrence of these complications cannot be ruled out, however they can become less likely. More importantly, it was anesthesiological management with high-dose opioid anesthetics which made prolonged ventilatory support of heart surgery patients necessary per se, and the time of extubation was already established intraoperatively [6-8]. By the late 1970s, however, sporadic case reports or series with small case numbers were published which pointed out the possibility of earlier extubation in heart surgery patients [3,9-12]. Interest in fast track protocols was rekindled because of a growing pressure on the health systems worldwide due to rapidly growing patient numbers, a steadily ageing patient population, increasing comorbidities and increasingly scarce resources. One possibility is the optimised use of available intensive care capacities by having several patients on one hospital bed daily. This requires efficient surgical planning, as well as a meticulous preoperative assessment of the patient population [1-4,13-22]. Besides shortening ICU occupancy times, which is undisputedly the limiting factor and the bottleneck in the care of heart surgery patients, the rising costs can be contained by shortening the overall hospitalisation period [16,19,20]. The precondition for introducing fast-track concepts was the continuous further development of existing surgical, cardiac technological, anesthesiological and postoperative management [1-4,7,13,23-25]. The introduction of short-acting substances such as propofol or remifentanil as well as inhalation anesthetics such as sevoflurane has contributed decisively to changing monitored anesthesia care. Calafiore [20] describes an increase in patient flow of about 15% in the ICU after the introduction of a fast-track concept without a negative impact on the quality of patient care, expressed as the incidence of postoperative morbidity or mortality. A further point is the fact that the number of postponed or cancelled operations decreases drastically due to the optimised use of limited intensive care capacities through fast-track programs.

“Fast track” however is not a concept with a rigid, clear definition, but rather a process for shortening any partial aspect of the established procedure. The term “fast track” includes the shortening of prolonged ventilatory support or even the immediate postoperative extubation of heart surgery patients, reducing ICU residence time or even direct transfer to an intermediate care unit, as well as reducing the hospitalisation time by already discharging the patient from hospital on the first postoperative day. The multiplicity of possible approaches embodied in different fast-track concepts substantially impedes comparison of the different publications in the literature. In the present study, the term “fast track“is understood to mean extubation of the patient on the day of surgery and immediate transfer to the IMC unit by 7.30 p.m. on the same day. The IMC unit has no facilities for invasive ventilation.

The goal of the retrospective study is to closely observe and describe the pre- and intraoperative parameters as well as the postoperative outcome of all fast-track patients over one full year. The risk variables for readmission to the intensive care unit are worked out in logistic regressions.

## Methods

The retrospectively recorded data of patients undergoing cardiac surgery within an 11-month period beginning in June 2010 at Goethe University in Frankfurt am Main, Germany, were entered in a database. The only requirement was the use of extracorporeal circulation (ECC).

Fast-track protocol anesthesia: After informing the patients and reviewing the patients’ findings, the cardiosurgical operation coordinator enrols the eligible patients for a fast-track protocol. Absolute contraindications are repeat interventions, aortic interventions with hypothermia, combined interventions involving more than 2 valve corrections.

Anesthesia management requires peripheral venous access, a radial artery catheter, a central venous catheter (right internal jugular vein), as well as a ventilation tube, a stomach tube and a bladder catheter. Anesthesia is induced using a balanced technique with sufentanil, disoprivan and rocuromium. Anesthesia is maintained with sevoflurane, rocuromium and remifentanil. Respiration is monitored routinely at fixed time intervals by blood gas analysis (BGA) measurements. Tranexamic acid is administered as a standard measure. Before connection to the extracorporeal circulation, every patient is given weight adapted intravenous heparin for full heparinisation. After weaning from the extracorporeal circulation (ECC) the effect of the heparin is antagonised with protamine and adjusted to normal values on the basis of the ACT measurement. Patients are transferred ventilated to the ICU where ECG, chest radiography and clinical laboratory tests as well as a blood gas analysis are immediately performed.

Fast-track protocol ICU: The blood gas analyses are repeated at hourly intervals, the usual laboratory parameters after 4 and 12 hours. Vital signs are monitored by continuous ECG recording, invasive measurements of blood pressure and central venous pressure (CVP) as well as monitoring of oxygen saturation. Standard pain medication comprises a combination of piritramide and a nonsteroidal anti-inflammatory drug (NSAID), e.g. metamizole or paracetamol. Criteria for extubation were: hemodynamic stability, bleeding <100 ml/h, 95% O2 saturation with FiO2 less than 0.5 and adequate patient response to questions. After extubation, the patients were transferred to the IMC unit at the latest by 7.30 p.m. on the day of surgery. In the IMC there was also continuous monitoring of vital signs, but there were no facilities for invasive ventilation.

Statistics: The data were transferred to the SPSS 20.0 program for statistical analysis. Fischer`s test and Student`s *t*-test were used to calculate statistical significances. P-values <0.05 are considered statistically significant. Multiple regressions are also performed for the validation of risk variables. 25 preoperative, 8 intraoperative and 25 postoperative variables were included for the calculation in the overall logistic regression model. The risk variables are evaluated in a univariate model for the endpoints “Readmission to or continued residence in ICU“and were finally included stepwise in multivariable, logistic models (cut-off: p value < 0,05). The following complications were subsumed under the heading of postoperative cardiac morbidity: new myocardial infarction, low cardiac output syndrome, multiorgan failure, cardiopulmonary resuscitation, implantation of an intra-aortic balloon pump (IABP). The concept of postoperative non-cardiac morbidity is defined by the following complications: intubation period longer than 24 hours, total reintubations, pneumonia, adult respiratory distress syndrome (ARDS), cerebrovascular stroke, postoperative renal failure, total rethoracotamies, sternal infection, sepsis. We calculated receiver operating characteristics (ROC) in order to describe a correlation of ICU re-admission and continuous variables. The area under the curve (AUC) with an associated 95% confidence interval (CI) was used as a measurement for the discriminating capacity. An ROC AUC value of 0.60-0.69 demonstrates a poor predictive value, 0.70-0.79 a moderate predictive value, 0.80-0.89 a good predictive value and 0.90-0.99 an excellent predictive value.

Conducting of the trial was verified by the Ethics Committee of the Johann Wolfgang Goethe University Frankfurt am Main. Patients were not informed in writing about the contents of the study and gave not their consent to participate. The reason for that, is that the procedure “fasttrack” was not randomized and is standard of care in our institution. Also patient data was extracted from the clinical database without any additional questionnaires. The extracted data was anonymous.

## Results

Within the 11-month observation period, 229 patients were preoperatively declared as potential fast- track patients. In fact, however, only 148 patients successfully completed the fast- track program. 36% of the patients either could not be transferred on the evening of the day of surgery (n = 77) or the patients had to be readmitted to the ICU after transfer during hospitalisation (n = 4). The commonest reasons were postoperative hemorrhage and respiratory problems. The demographic data and preoperative data of the readmitted patients compared to the fast-track patients differ only in the ASA and NYHA classification. All other values showed no significant differences (Table 1). Intraoperatively, a significant difference was observed both in the operation time and the perfusion time. After 274 minutes (mean + standard error) operation time or 142 min (mean + standard error) perfusion time, fast-track schedules should be terminated. In contrast, the ROC analysis of ICU-readmission and operation time demonstrates a poor predictive value (AUC 0,684). After this time there is a significant increase in the probability that the patients cannot leave the ICU on the day of surgery, or that they will be readmitted to the ICU during hospital treatment. These events must be avoided. 19 of 25 recorded postoperative parameters showed markedly significant differences in the groups of successful versus unsuccessful fast-track patients. Not differentiated with these outcome variables is whether this is a cause or consequence of readmission (Table 2). Major outcome events were combined in a regression model and tested first in a univariate and then in a multivariate model. As a risk variable, readmission to an intensive care unit is the main predictor of a major postoperative event (Table 3).

**Table 1:** Baseline and surgical characteristics

	**Fast-frack ICU**		**Readmitted fast track ICU**		**p**
	**N**	**%**	**N**	**%**	
	**148**	**64**	**81**	**36**	
Male	112	75,7	65	80,2	0,26
Main stem stenosis	38	25,7	27	33,3	0,14
Ejection fraction < 40%	19	12,8	16	19,8	0,11
Prior Percutanous Cardiac Intervention	62	41,9	35	43,2	0,47
ASA > 3	19	12,8	20	24,7	0,01
NYHA > 3	57	38	41	50,6	0,05
Hypertension	142	95	75	92,6	0,21
Pulmonary hypertension	10	6,8	11	13,6	0,07
Dyslipidaemia	109	73,6	64	79	0,23
Diabetes mellitus	40	27	20	24,7	0,41
Renal insufficiency	14	9,5	13	16	0,1
COPD	13	8,8	10	12,3	0,26
Smoker	25	16,9	9	11,1	0,16
Stroke	1	0,7	2	2,5	0,26
Carotis stenosis >50%	17	11,5	7	8,6	0,33
Urgent (need for operation within 24 h)	0	0	1	1,2	0,34
CABG	92	62,6	46	56,8	0,23
	Mean	Std	Mean	Std	P
Age	67	10,8	65,7	11,9	0,38
EuroSCORE I (additive)	4	2,2	4,1	2,6	0,63
BMI	27	3,3	28	4,4	0,56
CK	105	68	126	150	0,23
Operation_time	222	53	267	74	0,000
Perfusion time	102	34	130	49	0,000
Cross clamp time	63	28	79	38	0,000

**Table 2:** Postoperative characteristics and events

	**Fast-Track ICU**		**Readmitted Fast Track ICU**		**p**
	**N**	**%**	**N**	**%**	
	**148**	**64**	**81**	**36**	
Mesenteric complications	2	1,4	4	4,9	0,11
IABP	0	0	3	3,7	0,04
Low cardiac output*	1	0,7	9	11,1	0,0001
Mortality 30 d*	0	0	7	10,3	0,0001
Multi Organ Failure	0	0	4	4,9	0,01
Renal failure	1	0,7	9	11,1	0,0001
Psychosyndrome	0	0	8	9,9	0,0001
Resuscitation	1	0,7	7	8,6	0,003
Resp. insufficiency	8	5,4	10	12,3	0,06
Re-thoracotomy*	3	2	18	22	0,0001
Atrial fibrillation	48	32	25	30,9	0,4
Septic shock*	0	0	4	4,9	0,01
Wound healing disturbances*	6	4,1	11	13,6	0,07
Stroke*	1	0,7	2	2,5	0,13
Major postoperative event*	17	11,5	36	44,4	0,0001
Platelet transfusion	20	13,5	29	35,8	0,000
CK (U/l)	307	±26,9	412	±409	0,04
CK-MB (U/l)	31	±23,5	39	±27,1	0,04
Creatinine 1 po (mg/dl)	1,2	±0,65	1,39	±1,14	0,12
Time on ventilation (h)	1,95	±3,4	86,5	±34,5	0,03
Hospital stay (d)	10,3	±2,5	16,5	±16,3	0,001
ICU stay (d)	0	0±	63	±15	0,0001
Transfusion RBC (Unit)	1,44	±1,7	5,3	±9,1	0,0001
Transfusion FFP (Unit)	0,22	±0,74	1,54	±1,6	0,0001
Blood loss drainage (ml)	454	±358	529	507	0,2

**Table 3:** Factors identified as predictive of major postoperative events* by uni-and multivariate analysis

	**Univariate**			**Multivariate**		
	**p**	**HR**	**95% CI**	**p**	**HR**	**95% CI**
Readmission ICU	,001	,144	,057-,364	,001	,149	,069-,32
Prior Prior Percutanous Cardiac Intervention	,047	2,586	1,014-6,596	,02	,41	,195-,089
Ejection fraction < 40%	,055	2,733	,978-7,633	,01	3,07	1,23-7,57
Carotid stenosis > 50%	,103	3,193	,792-12,873			
Operation time >280 min	,106	,353	,100-1,248			
Smoker	,151	2,324	,736-7,338			
BMI > 30	,161	2,333	,714-7,622			
Main stem stenosis	,161	2,256	,724-7,031			
Dyslipidaemia	,218	,489	,156-1,528			
Pulmonary Hypertension	,219	2,572	,571-11,581			
Euroscore > 8	,227	,468	,137-1,603			
COPD	,252	,493	,147-1,653			
Perfusion time > 100 min	,291	1,899	,578-6,242			
CABG	,435	1,653	,468-5,841			
Diabetes mellitus	,560	,759	,300-1,917			
Hypertension	,737	1,388	,205-9,406			
Cross clamp time >60 min	,784	1,188	,346-4,087			
Age > 65 ys	,802	1,134	,424-3,036			
Male	,809	,875	,295-2,594			
ASA > 3	,864	1,118	,313-3,999			
Renal insufficiency	,915	1,070	,308-3,716			
NYHA > III	,945	1,034	,404-2,643			

(Major postoperative events* = low cardiac output, mortality, renal failure, re-thoracotomy, septic shock, wound healing disturbances, stroke.

## Discussion

The cardiac surgery patient population consecutively increases not only the follow-up costs, but also pushes available capacities up against increasingly narrow limits [14-18]. To meet the burgeoning medical expectations of this patient population in a situation of slowly growing or stagnating hospital resources and capacities, fast-track concepts have been gradually introduced into daily clinical practice. These concepts, which entail rapid postoperative extubation of cardiac surgery patients, short postoperative residence in complex, personnel and cost intensive monitoring units, as well as shortened postoperative hospitalisation periods, would have been inconceivable even several years ago. Only the optimal utilisation of available resources, such as the multiple daily use of an intensive care bed or the optimised design of surgery schedules including the fast-track concept can satisfy the growing demands and expectations of today’s heart surgery patients. Only considerable improvements in surgical, extracorporeal, anesthesiological and perioperative management have created the possibility of this fast-track approach [1-4,7,13,23-25]. The fast-track concept, however, is not subject to a rigid, uniform definition, but rather reflects the aspiration to transform established conventional techniques or procedures into modern patient management. One approach to the fast-track procedure is early postoperative extubation of patients. Publications from the 1990s declare patients with a postoperative ventilation period of less than 8 hours as a fast-track population [26]. The patients of the conventional treatment group, by contrast, were sedated in the established manner for longer than 12 hours postoperatively and accordingly received prolonged ventilatory support until the next morning. A further concept of the fast-track approach is shortening of the postoperative monitoring phase in the intensive care unit. But only by shortening the period of prolonged ventilatory support could the monitoring times also be consecutively shortened in the ICU [21,27]. While a study of Kogan [21] defines the postoperative transfer of patients from the ICU on the 1^st^ postoperative day as a fast-track population, in an article of Calafiore the patients of the fast-track population [20] were already transferred from the intensive care unit on the day of surgery. Since the fast-track concept has many facets, usually only a very limited comparison of published studies on the fast-track topic is possible. An additional hindrance is that most articles do not consider the entire spectrum of cardiac surgery patients but only cover a selected subgroup of the total patient pool for their fast-track protocols. Based on an optimal and realistic preoperative patient assessment, altogether 229 patients were included in a fast-track concept. An analysis of the demographic data and the preoperative risk factors of the fast-track patients yields a mean age of 67 ± 10 years. In the literature, the patients selected for the fast-track groups are a mean 59 ± 9 to 63 ± 10 years old and somewhat younger than the normal conventional population [7,19-21,28-30]. In addition, the percentage of female patients in the fast-track group is low with 24%. In other publications, the percentage of women among the fast-track patients is reported as 14–26, 4% and does not differ appreciably from the fast-track group studied here [19-21,28-30]. It must be assumed that, the fast-track patients in this study do not differ substantially in their demographic data from other studies. The high number of readmissions to the intensive care unit, however, is astonishing because a comparable study of Kogan et al. describes a readmission rate of only 3.3%. The reasons are again consistent with our own results; the predominant issues are postoperative hemorrhage and respiratory problems [21,31].

## Conclusion

The ASA > 3, NYHA class > 3 and operation time >267 minutes are independent predictors of fast track protocol failure. Therefore, parameters should be used more strictely in fast track patient selection and fast track termination.

## Abbreviations

ICU: Intensive care unit; IMC: Intermediate care unit; BMI: Body mass index; NYHA: New York heart association; ASA: American Society of Anesthesiologists, CABG: Coronary artery bypass graft; COPD: Chronic obstructive pulmonary disease; CK: Creatinin kinase; CVP: Central venous pressure; ECC: Extracorporeal circulation; BGA: Blood gas analysis; ECG: Electrocardiogram; ACT: Activated clotting time; NSAID: Nonsteroidal anti-inflammatory drug; ABP: Intra-aortic balloon pump; ARDS: Adult respiratory distress syndrome; RBC: Red blood cells; FFP: Fresh frozen plasma.

## Competing interests

The authors declare that they have no competing interests.

## Authors’ contribution

AHK: Conceived the study and designed the study, analysis and interpretation of data and coordination and wrote the manuscript. PH: Participated in the design of the study, data recruitment, performed the statistical analysis and helped to draft the final manuscript. CR: Participated for the intraoperative, anaesthesiology regime, have been involved in drafting the manuscript, revising it critically for important intellectual content. TB: Participated for the postoperative, anaesthesiology regime, have been involved in drafting the manuscript, revising it critically for important intellectual content. AZ: Participated for the preoperative fast track regime, have been involved in drafting the manuscript, revising it critically for important intellectual content. AM Conceived the study, coordination and revising it critically for important intellectual content. All authors read and approved the final manuscript.
